# Supplementary material for: Fostering temporal crop diversification to reduce pesticide use
Source: Nat Commun. 2023 Nov 16;14:7416. doi: 10.1038/s41467-023-43234-x (PMC10654721; doi:10.1038/s41467-023-43234-x)
Supplement: Supplementary file 1 — Supplementary Information [file 41467_2023_43234_MOESM1_ESM.pdf]

# Supplementary Information

## Fostering temporal crop diversification to reduce pesticide use

Maé Guinet[1a*], Guillaume Adeux[1a], Stéphane Cordeau[1], Emeric Courson[1], Romain Nandillon[1], Yaoyun Zhang[1], Nicolas Munier-Jolain[1]

[1] Agroécologie, Institut Agro, INRAE, Univ. Bourgogne, Univ. Bourgogne Franche-Comté, F-21000 Dijon, France

[a] These authors contributed equally

*Corresponding author: mae.guinet@inrae.fr

### Table of contents

Supplementary Tables ........................................................................................................................ 1
Supplementary Figures ...................................................................................................................... 9

Supplementary Tables

**Supplementary Table 1: List of all the Generalized linear mixed effect models (GLMMs) fitted in this study**. Formulas follow the syntax of the function and package used for analysis (i.e. R function glmmTMB() from the {glmmTMB} package). "*" denotes main and interaction effects, ":" denotes interaction effects only. All models were fitted with a Tweedie error distribution and a log link function. All random effects are random intercepts. Models 1-3 were fitted at the cropping system scale for each available time point (time point referring to either the two-to-three-year average provided by farmers upon entry in the network or the subsequent annual descriptions) whereas Models 4-15 were fitted at the crop scale (values were averaged when a given crop was grown over multiple fields of a given cropping system at a given time point). The results of Type III analysis of deviance (two-tailed Wald chisquare tests) are shown and highlight the effect of i) climatic regions on crop diversity indicators (models 1-3), ii) crop on crop diversity indicators while controlling for climatic region effects (models 4-6), iii) crop on crop pesticide use while controlling for climatic region effects (models 7-9) and iv) diversity indicators on crop pesticide use (TFI) while accounting for climatic region and crop identity effects (models 10-15). Significant effects ($p \leq 0.05$) are in bold. df: degrees of freedom.

| Model | Response | Random effects | Predictors | $X^2$ | df | p.value |
|---|---|---|---|---|---|---|
| 1 | functionnal diversity (n=3761) | (1\|cs code) + (1\|description period) + (1\|climatic region:description period) + (1\|climatic region:soil) | climatic region | 12.00 | 5 | **0.03477** |
| 2 | taxonomic diversity (n=3761) | (1\|cs code) + (1\|description period) + (1\|climatic region:description period) + (1\|climatic region:soil) | climatic region | 40.84 | 5 | **1.011e-07** |
| 3 | species diversity (n=3761) | (1\|cs code) + (1\|description period) + (1\|climatic region:description period) + (1\|climatic region:soil) | climatic region | 24.44 | 5 | **0.0001789** |
| 4 | functionnal diversity (n=14456) | (1\|description period) + (1\|crop:description period) + (1\|climatic region:description period) + (1\|climatic region:crop) + (1\|climatic region:soil) +(1\|crop :climatic region:description period) | climatic region | 6.17 | 5 | 0.2897 |
| | | | crop | 316.18 | 15 | **< 2e-16** |
| 5 | taxonomic diversity (n=14456) | (1\|description period) + (1\|crop:description period) + (1\|climatic region:description period) + (1\|climatic region:crop) + (1\|climatic region:soil) +(1\|crop:climatic region:description period) | climatic region | 6.00 | 5 | 0.3082 |
| | | | crop | 564.46 | 15 | **< 2e-16** |
| 6 | species diversity (n=14456) | (1\|description period) + (1\|crop:description period) + (1\|climatic region:description period) + (1\|climatic region:crop) + (1\|climatic region:soil) +(1\|crop:climatic region:description period) | climatic region | 12.02 | 5 | **0.03452** |
| | | | crop | 109.61 | 15 | **< 2e-16** |

| Model | Response | Random effects | Predictors | X² | df | p.value |
|---|---|---|---|---|---|---|
| 7 | crop total TFI (n=14456) | (1\|description period)<br>+ (1\|cs code)<br>+ (1\|cs code: crop)<br>+ (1\|cs code:description period)<br>+(1\|crop:description period)<br>+ (1\|climatic region:description period)<br>+ (1\|climatic region:crop)<br>+ (1\|climatic region:soil)<br>+(1\|crop:climatic region:description period) | climatic region<br>crop | 16.54<br>1294.74 | 5<br>15 | **0.005451**<br>**< 2.2e-16** |
| 8 | crop herbicide TFI (n=14456) | (1\|description period)<br>+ (1\|cs code)<br>+ (1\|cs code: crop)<br>+ (1\|cs code:description period)<br>+ (1\|crop:description period)<br>+ (1\|climatic region:description period)<br>+ (1\|climatic region:crop)<br>+ (1\|climatic region:soil)<br>+(1\|crop:climatic region:description period) | climatic region<br>crop | 14.66<br>992.76 | 5<br>15 | **0.0119**<br>**< 2.2e-16** |
| 9 | crop fungicide TFI (n=14456) | (1\|description period)<br>+ (1\|cs code)<br>+ (1\|cs code: crop)<br>+ (1\|cs code:description period)<br>+(1\|crop:description period)<br>+ (1\|climatic region:description period)<br>+ (1\|climatic region:crop)<br>+ (1\|climatic region:soil)<br>+(1\|crop:climatic region:description period) | climatic region<br>crop | 13.14<br>727.09 | 5<br>15 | **0.02207**<br>**< 2e-16** |
| 10 | crop insecticide TFI (n=14456) | (1\|description period)<br>+ (1\|cs code)<br>+ (1\|cs code: crop)<br>+ (1\|cs code:description period)<br>+(1\|crop:description period)<br>+ (1\|climatic region:description period)<br>+ (1\|climatic region:crop)<br>+ (1\|climatic region:soil)<br>+(1\|crop:climatic region:description period) | climatic region<br>crop | 9.54<br>301.25 | 5<br>15 | 0.08927<br>**< 2e-16** |

| Model | Response | Random effects | Predictors | X² | df | p.value |
|---|---|---|---|---|---|---|
| 11 | crop total TFI (n=12812) | (1\|description period) + (1\|cs code) + (1\|cs code: crop) + (1\|cs code:description period) +(1\|crop:description period) + (1\|climatic region:description period) + (1\|climatic region:crop) + (1\|climatic region:soil) +(1\|crop:climatic region:description period) | climatic region | 8.51 | 5 | 0.1300854 |
| | | | crop | 550.08 | 11 | **< 2.2e-16** |
| | | | functionnal diversity | 6.57 | 1 | **0.0103855** |
| | | | taxonomic diversity | 13.82 | 1 | **0.0002008** |
| | | | functionnal diversity: taxonomic diversity | 0.30 | 1 | 0.5849230 |
| | | | cover crop frequency | 24.34 | 1 | **8.057e-07** |
| | | | crop:functionnal diversity | 56.83 | 11 | **3.573e-08** |
| | | | crop:taxonomic diversity | 25.97 | 11 | **0.0065533** |
| 12 | crop herbicide TFI (n=12812)) | (1\|description period) + (1\|cs code) + (1\|cs code: crop) + (1\|cs code:description period) + (1\|crop:description period) + (1\|climatic region:description period) + (1\|climatic region:crop) + (1\|climatic region:soil) +(1\|crop:climatic region:description period) | climatic region | 7.60 | 5 | 0.1798196 |
| | | | crop | 307.17 | 11 | **< 2.2e-16** |
| | | | functionnal diversity | 0.00 | 1 | 0.9791366 |
| | | | taxonomic diversity | 5.38 | 1 | **0.0203128** |
| | | | functionnal diversity: taxonomic diversity | 0.01 | 1 | 0.9130258 |
| | | | cover crop frequency | 9.13 | 1 | **0.0025127** |
| | | | crop:functionnal diversity | 73.79 | 11 | **2.31e-11** |
| | | | crop:taxonomic diversity | 36.60 | 11 | **0.0001342** |
| 13 | crop herbicide TFI (n=12812)) | (1\|description period) + (1\|cs code) + (1\|cs code: crop) + (1\|cs code:description period) +(1\|crop:description period) + (1\|climatic region:description period) + (1\|climatic region:crop) + (1\|climatic region:soil) +(1\|crop:climatic region:description period) | climatic region | 7.34 | 5 | 0.196525 |
| | | | crop | 304.18 | 11 | **< 2.2e-16** |
| | | | sowing diversity | 7.67 | 1 | **0.005613** |
| | | | taxonomic diversity | 0.57 | 1 | 0.452194 |
| | | | cover crop frequency | 8.93 | 1 | **0.002804** |
| | | | crop:sowing diversity | 29.73 | 11 | **0.001749** |
| | | | crop:taxonomic diversity | 28.54 | 11 | **0.002670** |
| 14 | crop fungicide TFI (n=9419) | (1\|description period) + (1\|cs code) + (1\|cs code: crop) + (1\|cs code:description period) +(1\|crop:description period) + (1\|climatic region:description period) + (1\|climatic region:crop) + (1\|climatic region:soil) +(1\|crop:climatic region:description period) | climatic region | 11.65 | 5 | **0.039937** |
| | | | crop | 485.76 | 8 | **< 2.2e-16** |
| | | | functionnal diversity | 6.13 | 1 | **0.013293** |
| | | | taxonomic diversity | 12.76 | 1 | **0.000355** |
| | | | functionnal diversity: taxonomic diversity | 1.60 | 1 | 0.206508 |
| | | | cover crop frequency | 3.55 | 1 | 0.059530 |
| | | | crop:functionnal diversity | 23.15 | 8 | **0.003179** |
| 15 | crop insecticide TFI (n=2950) | (1\|description period) + (1\|cs code) + (1\|cs code: crop) + (1\|cs code:description period) +(1\|crop:description period) + (1\|climatic region:description period) + (1\|climatic region:crop) + (1\|climatic region:soil) +(1\|crop:climatic region:description period) | climatic region | 6.70 | 5 | 0.243592 |
| | | | crop | 54.77 | 3 | **7.674e-12** |
| | | | functionnal diversity | 0.17 | 1 | 0.682793 |
| | | | taxonomic diversity | 0.08 | 1 | 0.772835 |
| | | | functionnal diversity: taxonomic diversity | 0.85 | 1 | 0.356266 |
| | | | cover crop frequency | 4.38 | 1 | **0.036305** |
| | | | crop:functionnal diversity | 8.20 | 3 | **0.042013** |
| | | | crop:taxonomic diversity | 12.74 | 3 | **0.005226** |

**Supplementary Table 2: List of crops present in the dataset (vernacular and latin names) and their membership to different botanical families and sowing periods.** Crops are ranked according to their average proportion across all rotations.

| Botanical family | Crop | Latin name | Group | Sowing period | Average proportion across all rotations (%) |
|---|---|---|---|---|---|
| Poaceae | Winter wheat | *Triticum aestivum* | 1 | winter | 30.6598 |
| Poaceae | Maize | *Zea mays* | 1 | summer | 21.6442 |
| Brassicaceae | Oilseed rape | *Brassica napus* | 3 | autumn | 8.5052 |
| Poaceae | Winter barley | *Hordeum vulgare* | 1 | winter | 7.4852 |
| Poaceae/Fabaceae | Grassland | | 7 | perenial | 7.2078 |
| Asteraceae | Sunflower | *Helianthus annuus* | 4 | summer | 3.1384 |
| Poaceae | Spring barley | *Hordeum vulgare* | 1 | spring | 3.0047 |
| Amaranthaceae | Sugarbeet | *Beta vulgaris subsp. vulgaris* | 6 | spring | 2.6047 |
| Fabaceae | Alfalfa | *Medicago sativa* | 2 | perennial | 2.1606 |
| Poaceae/Fabaceae | Winter cereal-legume mixture | | 7 | winter | 2.0548 |
| Poaceae | Ryegrass | *Lolium spp.* | 1 | winter | 1.7757 |
| Poaceae | Winter durum wheat | *Triticum durum* | 1 | winter | 1.6804 |
| Poaceae | Triticale | *x Triticosecale* | 1 | winter | 1.5186 |
| Fabaceae | Spring pea | *Pisum sativum* | 2 | spring | 1.1668 |
| Fabaceae | Soybean | *Glycine max* | 2 | summer | 1.0092 |
| Solanaceae | Potato | *Solanum tuberosum* | 10 | summer | 0.9273 |
| Linaceae | Spring flax | *Linum usitatissimum* | 9 | spring | 0.7517 |
| Poaceae | Sorghum | *Sorghum bicolor* | 1 | summer | 0.4483 |
| Fabaceae | Spring fababean | *Vicia faba* | 2 | spring | 0.4310 |
| Fabaceae | Winter pea | *Pisum sativum* | 2 | winter | 0.4017 |
| Fabaceae | Winter Fababean | *Vicia faba* | 2 | winter | 0.4009 |
| Fabaceae | Lentils | *Lens culinaris* | 2 | spring | 0.3251 |
| Brassicaceae | Oilseed rape with companion plants | *Brassica napus* | 3 | autumn | 0.2436 |
| Fabaceae | Clover meadow | *Trifolium sp.* | 2 | perennial | 0.2199 |
| Fabaceae | Bean | *Phaseolus vulgaris* | 2 | summer | 0.2109 |
| Poaceae | Spring aot | *Avena sativa* | 1 | spring | 0.2059 |
| Poaceae | Spring wheat | *Triticum aestivum* | 1 | spring | 0.1954 |
| Cannabaceae | Hemp | *Cannabis sativa* | 13 | summer | 0.1824 |
| Fabaceae | Spring garden pea | *Pisum sativum* | 2 | spring | 0.1635 |
| Polygonaceae | Buckwheat | *Fagopyrum esculentum* | 11 | summer | 0.1589 |
| Linaceae | Winter flax | *Linum usitatissimum* | 9 | winter | 0.1470 |
| Poaceae | Winter oat | *Avena sativa* | 1 | winter | 0.1378 |
| Poaceae | Winter rye | *Secale cereale* | 1 | winter | 0.1226 |
| Amaryllidaceae | Garlic | *Allium sativum* | 8 | spring | 0.1142 |
| Poaceae | Common millet | *Panicum miliaceum* | 1 | summer | 0.1110 |
| Apiaceae | Carrot | *Daucus carota subsp. sativus* | 5 | spring | 0.0971 |
| Amaranthaceae | Forage beet | *Beta vulgaris* | 6 | spring | 0.0952 |
| Fabaceae | Winter garden pea | *Pisum sativum* | 2 | winter | 0.0772 |
| Brassicaceae | Winter forage oilseed rape | *Brassica napus* | 3 | autumn | 0.0740 |

| Botanical family | Crop | Latin name | Group | Sowing period | Average proportion across all rotations (%) |
|---|---|---|---|---|---|
| Fabaceae | Chickpea | *Cicer arietinum* | 2 | summer | 0.0715 |
| Poaceae | Orchardgrass | *Dactylis sp.* | 1 | perennial | 0.0700 |
| Poaceae | Cereal meadow | | 1 | perennial | 0.0679 |
| Poaceae | Winter cereal mixture | | 1 | winter | 0.0620 |
| Amaranthaceae | Chicory | *Cichorium intybus* | 6 | spring | 0.0617 |
| Brassicaceae | Brown mustard | *Brassica juncea* | 3 | autumn | 0.0579 |
| Poaceae | Spelt | *Triticum spelta* | 1 | winter | 0.0579 |
| Amaryllidaceae | Onions | *Allium cepa* | 8 | spring | 0.0558 |
| Poaceae/Fabaceae | Spring cereal-legumes mixture | | 7 | spring | 0.0534 |
| Fabaceae | Winter vetch | *Vicia sp.* | 2 | winter | 0.0452 |
| Fabaceae | Winter forage pea | *Pisum sativum* | 2 | winter | 0.0437 |
| Papaveraceae | Poppy | *Papaver somniferum* | 12 | spring | 0.0436 |
| Fabaceae | Legume meadow | | 2 | perennial | 0.0416 |
| Diverse | Diverse | | 14 | spring | 0.0302 |
| Fabaceae | Annual clover | *Trifolium sp.* | 2 | spring | 0.0300 |
| Fabaceae | Spring forage pea | *Pisum sativum* | 2 | spring | 0.0240 |
| Poaceae | Foxtail millet | *Setaria italica subsp. moharia* | 1 | summer | 0.0216 |
| Poaceae | Fescue meadow | *Festuca sp.* | 1 | perennial | 0.0204 |
| Amaranthaceae | Spinach | *Spinacia oleracea* | 6 | spring | 0.0178 |
| Fabaceae | Winter legume mixture | | 2 | winter | 0.0168 |
| Fabaceae | Winter Lupin | *Lupinus albus* | 2 | winter | 0.0156 |
| Poaceae | Brome meadow | *Bromus sp.* | 1 | perennial | 0.0148 |
| Fabaceae | Spring vetch | *Vicia sp.* | 2 | spring | 0.0138 |
| Fabaceae | Sainfoin meadow | *Onobrychis viciifolia* | 2 | perennial | 0.0136 |
| Fabaceae | Spring lupin | *Lupinus albus* | 2 | spring | 0.0125 |
| Poaceae | Spring durum wheat | *Triticum durum* | 1 | spring | 0.0124 |
| Hydrophyllaceae | Lacy phacelia | *Phacelia tanacetifolia* | 14 | spring | 0.0117 |
| Poaceae | Spring barley with companion plants | *Hordeum vulgare* | 1 | spring | 0.0114 |
| Brassicaceae | Spring oilseed rape | *Brassica napus* | 3 | spring | 0.0097 |
| Brassicaceae | Cabbage | *Brassica oleracea* | 3 | autumn | 0.0097 |
| Poaceae | Spring cereal-oilseeds mixture | | 1 | spring | 0.0089 |
| Apiaceae | Coriander | *Coriandrum sativum* | 5 | spring | 0.0082 |
| Fabaceae | Trefoil meadow | *Medicago lupulina* | 2 | perennial | 0.0076 |
| Brassicaceae | Radish | *Raphanus sativus* | 3 | autumn | 0.0075 |
| Poaceae | Winter cereal-oilseeds mixture | | 1 | winter | 0.0073 |
| Poaceae | Summer cereal mixture | | 1 | summer | 0.0066 |
| Asteraceae | Sunflower with companion plants | *Helianthus annuus* | 4 | summer | 0.0065 |
| Amaranthaceae | Quinoa | *Chenopodium quinoa* | 6 | spring | 0.0064 |
| Apiaceae | Parsley | *Petroselinum crispum* | 5 | spring | 0.0064 |
| Poaceae | Spring rye | *Secale cereale* | 1 | spring | 0.0057 |
| Poaceae | Miscanthus | *Miscanthus x giganteus* | 1 | perennial | 0.0057 |

| Botanical family | Crop | Latin name | Group | Sowing period | Average proportion across all rotations (%) |
|---|---|---|---|---|---|
| Brassicaceae | Autumn oilseed mixture | | 3 | autumn | 0.0053 |
| Brassicaceae | Turnip | *Brassica rapa subsp. rapa* | 3 | autumn | 0.0044 |
| Asteraceae | Summer oilseed-legume mixture | | 4 | summer | 0.0040 |
| Fabaceae | Spring legume mixture | | 2 | spring | 0.0033 |
| Amaryllidaceae | Chive | *Allium schoenoprasum* | 8 | spring | 0.0033 |
| Brassicaceae | Camelina | *Camelina sativa* | 3 | summer | 0.0032 |
| Apiaceae | Parsnip | *Pastinaca sativa* | 5 | spring | 0.0027 |
| Fabaceae | Black medick | *Medicago lupulina* | 2 | perennial | 0.0026 |
| Brassicaceae | Forage radish | *Raphanus sativus* | 3 | autumn | 0.0019 |
| Asteraceae | Safflower | *Carthamus tinctorius* | 4 | summer | 0.0018 |
| Fabaceae | Fenugreek | *Trigonella foenum-graecum* | 2 | summer | 0.0017 |
| Apiaceae | Anise | *Pimpinella anisum* | 5 | spring | 0.0017 |
| Poaceae/Fabaceae | Summer cereal-legumes mixture | | 7 | summer | 0.0017 |
| Asteraceae | Chamomile | *Matricaria chamomilla* | 4 | spring | 0.0017 |
| Lamiaceae | Common thyme | *Thymus vulgaris* | 14 | spring | 0.0017 |
| Asteraceae | Salsify | *Tragopogon porrifolius* | 4 | summer | 0.0017 |
| Brassicaceae | Spring forage oilseed rape | *Brassica napus* | 3 | spring | 0.0013 |
| Lamiaceae | Chia | *Salvia hispanica* | 14 | spring | 0.0012 |
| Poaceae | Spring cereal mixture | | 1 | spring | 0.0011 |
| Lamiaceae | Basil | *Ocimum basilicum* | 14 | spring | 0.0006 |
| Fabaceae | Vetchling | *Lathyrus sp.* | 2 | summer | 0.0006 |
| Poaceae | Canary grass | *Phalaris canariensis* | 1 | summer | 0.0006 |
| Poaceae | Common millet with companion plants | *Panicum miliaceum* | 1 | summer | 0.0002 |

**Supplementary Table 3:** Effect sizes and significance of slopes of crop diversity indicators (functional and taxonomic) on pesticide use (total and per type: herbicides, fungicides, and insecticides, all assessed with the Treatment Frequency Index (TFI), which quantifies the number of applications at the full recommended dose) for the main crops in which pesticides are commonly applied. Crops with null to low pesticide use (i.e. less than 0.7 for total pesticide use, less than 0.5 for herbicide, less than 0.1 for fungicide and less than 0.2 for insecticide) were not included in these analyses. Significance of slopes was assessed with a two-tailed Wald test, which tests the coefficients (i.e. slopes of diversity indicators for each crop) against zero. Degrees of freedom (df) are labelled as "Inf" (for infinite) in the case of asymptotic results (that is, when estimates are tested against the standard normal distribution – *z* tests – rather than the *t* distribution). SE: standard error; asymp.LCL: asymptotic lower confidence limit; asymp.UCL: asymptotic upper confidence limit.

| Response | Predictor | Crop | slope | SE | df | asymp.LCL | asymp.UCL | z.ratio | p.value |
|---|---|---|---|---|---|---|---|---|---|
| Total TFI | Functional diversity | Soybean | -0.088 | 0.046 | Inf | -0.178 | 0.003 | -1.903 | 0.0572 |
| | | Sunflower | -0.075 | 0.026 | Inf | -0.126 | -0.023 | -2.838 | **0.0045** |
| | | Maize | -0.071 | 0.017 | Inf | -0.105 | -0.037 | -4.112 | **< 0.0001** |
| | | Spring barley | -0.027 | 0.024 | Inf | -0.074 | 0.019 | -1.142 | 0.2536 |
| | | Triticale | -0.046 | 0.042 | Inf | -0.128 | 0.035 | -1.112 | 0.2663 |
| | | Winter durum wheat | 0.042 | 0.031 | Inf | -0.018 | 0.103 | 1.369 | 0.1712 |
| | | Spring pea | -0.016 | 0.030 | Inf | -0.075 | 0.043 | -0.521 | 0.6026 |
| | | Winter barley | 0.015 | 0.017 | Inf | -0.018 | 0.049 | 0.893 | 0.3716 |
| | | Winter wheat | 0.022 | 0.012 | Inf | -0.002 | 0.046 | 1.782 | 0.0746 |
| | | Oilseed rape | -0.018 | 0.016 | Inf | -0.049 | 0.012 | -1.183 | 0.2367 |
| | | Sugar beet | -0.081 | 0.022 | Inf | -0.124 | -0.038 | -3.704 | **0.0002** |
| | | Potato | 0.030 | 0.025 | Inf | -0.019 | 0.078 | 1.204 | 0.2289 |
| | Taxonomic diversity | Soybean | -0.023 | 0.050 | Inf | -0.121 | 0.076 | -0.450 | 0.6515 |
| | | Sunflower | -0.062 | 0.032 | Inf | -0.125 | 0.001 | -1.922 | 0.0548 |
| | | Maize | -0.051 | 0.016 | Inf | -0.083 | -0.019 | -3.101 | **0.0019** |
| | | Spring barley | -0.046 | 0.028 | Inf | -0.101 | 0.009 | -1.653 | 0.0984 |
| | | Triticale | 0.018 | 0.037 | Inf | -0.054 | 0.090 | 0.494 | 0.6214 |
| | | Winter durum wheat | -0.001 | 0.040 | Inf | -0.080 | 0.078 | -0.025 | 0.9793 |
| | | Spring pea | -0.069 | 0.034 | Inf | -0.136 | -0.003 | -2.057 | **0.0397** |
| | | Winter barley | -0.009 | 0.017 | Inf | -0.043 | 0.025 | -0.540 | 0.5891 |
| | | Winter wheat | -0.019 | 0.013 | Inf | -0.045 | 0.007 | -1.460 | 0.1441 |
| | | Oilseed rape | -0.072 | 0.018 | Inf | -0.107 | -0.037 | -4.014 | **0.0001** |
| | | Sugar beet | -0.005 | 0.033 | Inf | -0.070 | 0.060 | -0.157 | 0.8744 |
| | | Potato | -0.179 | 0.049 | Inf | -0.274 | -0.083 | -3.677 | **0.0002** |

| Response | Predictor | Crop | slope | SE | df | asymp.LCL | asymp.UCL | z.ratio | p.value |
|---|---|---|---|---|---|---|---|---|---|
| Herbicide TFI | Functional diversity | Soybean | -0.123 | 0.047 | Inf | -0.214 | -0.031 | -2.631 | **0.0085** |
| | | Sunflower | -0.073 | 0.030 | Inf | -0.132 | -0.014 | -2.434 | **0.0149** |
| | | Maize | -0.044 | 0.019 | Inf | -0.082 | -0.006 | -2.269 | **0.0234** |
| | | Spring barley | -0.038 | 0.029 | Inf | -0.096 | 0.019 | -1.299 | 0.1950 |
| | | Triticale | 0.033 | 0.049 | Inf | -0.062 | 0.129 | 0.682 | 0.4939 |
| | | Winter durum wheat | 0.073 | 0.038 | Inf | -0.002 | 0.147 | 1.898 | 0.0578 |
| | | Spring pea | 0.068 | 0.039 | Inf | -0.008 | 0.144 | 1.746 | 0.0805 |
| | | Winter barley | 0.030 | 0.021 | Inf | -0.011 | 0.071 | 1.427 | 0.1537 |
| | | Winter wheat | 0.063 | 0.015 | Inf | 0.034 | 0.092 | 4.205 | **< 0.0001** |
| | | Oilseed rape | 0.063 | 0.020 | Inf | 0.023 | 0.103 | 3.109 | **0.0019** |
| | | Sugar beet | -0.079 | 0.025 | Inf | -0.129 | -0.029 | -3.117 | **0.0018** |
| | | Potato | 0.031 | 0.040 | Inf | -0.047 | 0.110 | 0.784 | 0.4331 |
| | Taxonomic diversity | Soybean | -0.098 | 0.052 | Inf | -0.200 | 0.004 | -1.885 | 0.0596 |
| | | Sunflower | -0.040 | 0.036 | Inf | -0.111 | 0.031 | -1.113 | 0.2648 |
| | | Maize | -0.033 | 0.019 | Inf | -0.070 | 0.003 | -1.790 | 0.0736 |
| | | Spring barley | -0.070 | 0.034 | Inf | -0.137 | -0.003 | -2.033 | **0.0421** |
| | | Triticale | 0.090 | 0.044 | Inf | 0.005 | 0.176 | 2.072 | **0.0381** |
| | | Winter durum wheat | 0.027 | 0.049 | Inf | -0.068 | 0.122 | 0.556 | 0.5781 |
| | | Spring pea | 0.010 | 0.043 | Inf | -0.073 | 0.094 | 0.245 | 0.8065 |
| | | Winter barley | 0.005 | 0.021 | Inf | -0.036 | 0.047 | 0.241 | 0.8092 |
| | | Winter wheat | -0.001 | 0.016 | Inf | -0.033 | 0.031 | -0.072 | 0.9439 |
| | | Oilseed rape | -0.003 | 0.024 | Inf | -0.049 | 0.043 | -0.131 | 0.8946 |
| | | Sugar beet | 0.034 | 0.039 | Inf | -0.041 | 0.110 | 0.885 | 0.3764 |
| | | Potato | -0.311 | 0.072 | Inf | -0.452 | -0.170 | -4.330 | **< 0.0001** |
| Fungicide TFI | Functional diversity | Spring barley | -0.095 | 0.039 | Inf | -0.172 | -0.019 | -2.439 | **0.0147** |
| | | Triticale | -0.071 | 0.070 | Inf | -0.208 | 0.067 | -1.009 | 0.3131 |
| | | Winter durum wheat | 0.016 | 0.048 | Inf | -0.078 | 0.111 | 0.338 | 0.7351 |
| | | Spring pea | -0.066 | 0.048 | Inf | -0.160 | 0.028 | -1.384 | 0.1663 |
| | | Winter barley | -0.007 | 0.027 | Inf | -0.060 | 0.046 | -0.266 | 0.7899 |
| | | Winter wheat | -0.025 | 0.018 | Inf | -0.061 | 0.011 | -1.386 | 0.1657 |
| | | Oilseed rape | 0.025 | 0.027 | Inf | -0.028 | 0.078 | 0.937 | 0.3487 |
| | | Sugar beet | -0.139 | 0.035 | Inf | -0.206 | -0.071 | -4.017 | **0.0001** |
| | | Potato | 0.005 | 0.029 | Inf | -0.053 | 0.063 | 0.176 | 0.8606 |
| Insecticide TFI | Functional diversity | Spring pea | -0.098 | 0.088 | Inf | -0.270 | 0.073 | -1.123 | 0.2614 |
| | | Oilseed rape | -0.055 | 0.040 | Inf | -0.133 | 0.022 | -1.396 | 0.1628 |
| | | Sugar beet | -0.050 | 0.084 | Inf | -0.215 | 0.115 | -0.593 | 0.5529 |
| | | Potato | 0.280 | 0.117 | Inf | 0.050 | 0.509 | 2.384 | **0.0171** |
| | Taxonomic diversity | Spring pea | -0.354 | 0.100 | Inf | -0.550 | -0.158 | -3.545 | **0.0004** |
| | | Oilseed rape | -0.074 | 0.048 | Inf | -0.167 | 0.020 | -1.546 | 0.1222 |
| | | Sugar beet | 0.060 | 0.123 | Inf | -0.181 | 0.300 | 0.487 | 0.6265 |
| | | Potato | 0.291 | 0.191 | Inf | -0.084 | 0.666 | 1.520 | 0.1286 |

## Supplementary Figures

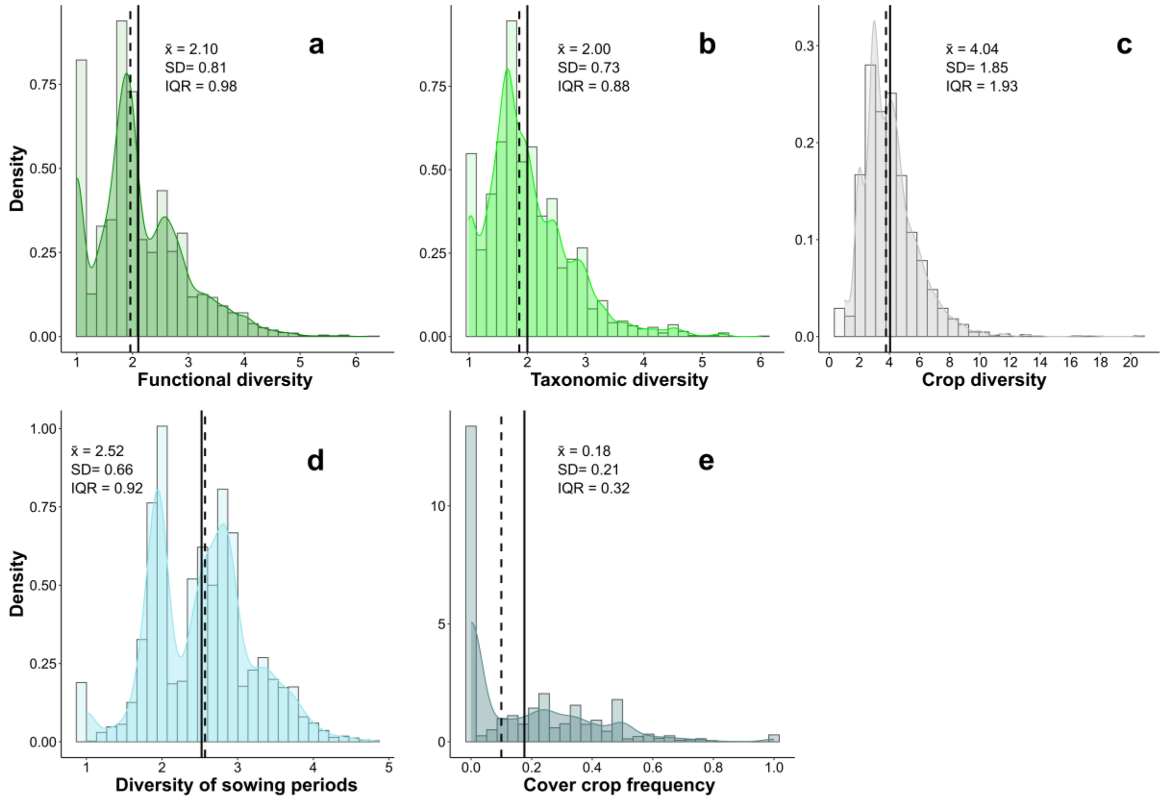


**Supplementary Figure 1: Probability density of the five crop diversity indicators considered in this study. a: Functional diversity, b: Taxonomic diversity**, **c: Crop diversity**, **d: diversity of sowing periods** and **e: Cover crops frequency.** One data point (N=3761) represents the value of a diversity indicator computed for a given cropping system at a given time point (time point referring to either the two-to-three-year average provided by farmers upon entry in the network or the subsequent annual descriptions). Kernel bandwidth was chosen using Silverman's rule-of-thumb method. Probability density was estimated with gaussian kernel method and describes the probability densities associated with diversity indicators. Vertical solid lines represent the mean. Vertical dashed lines represent the median. x̄ = mean; SD= standard deviation; IQR= Interquartile range

Crop taxonomic diversity, crop functional diversity, the interaction between crop taxonomic and functional diversity (i.e. crop diversity) and diversity of sowing periods averaged 2.0±0.7, 2.1±0.8, 4.0±1.8 and 2.5±0.7, respectively, across all 3761 combinations of cropping system and time points including at least one of the 16 main crops of the dataset. Cover crop frequency averaged 0.18 (±0.20) across these same cropping systems

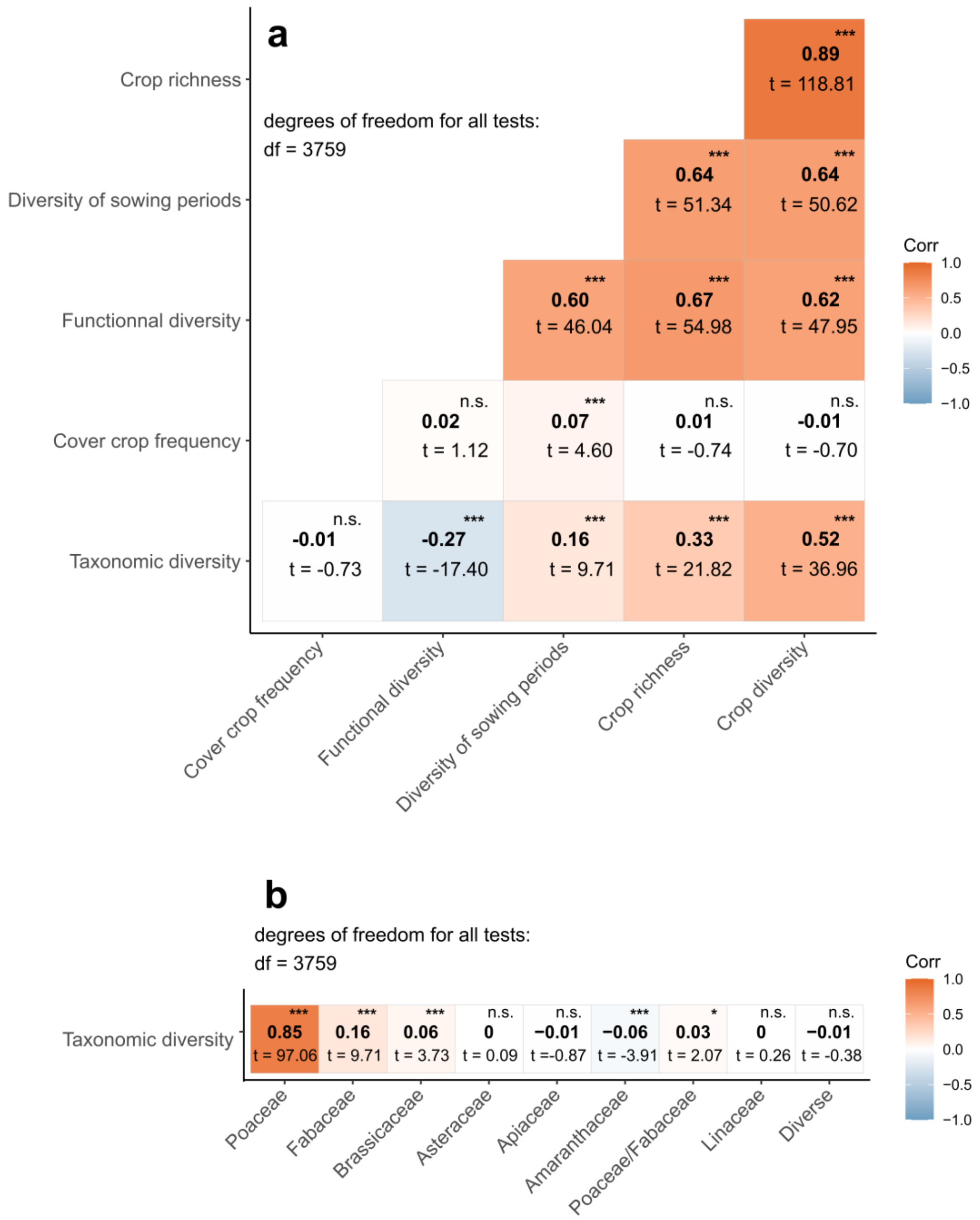


**Supplementary Figure 2: Pearson's correlation matrix between a) crop diversity indicators and b) crop Taxonomic diversity and the diversity of crops within a botanical family**. Only botanical families with more than one crop were included. Positive and negative correlations are displayed in orange and blue, respectively. Color intensity is proportional to the strength of the correlation coefficients. *** indicates significant correlations ($p < 0.001$). n.s.: non-significant.

Pearson's product moment correlations between the four crop diversity indicators were all significant and absolute strength of association exceeded 0.6 between crop functional diversity, diversity of sowing periods and crop diversity. Similarly, all four crop diversity indicators were significantly correlated ($p<0.001$) with crop richness and strength of association exceeded 0.6 for crop functional diversity, diversity of sowing periods, and crop diversity. Cover crop was significantly correlated to diversity of sowing periods, albeit with a weak strength of association ($r$=0.07).

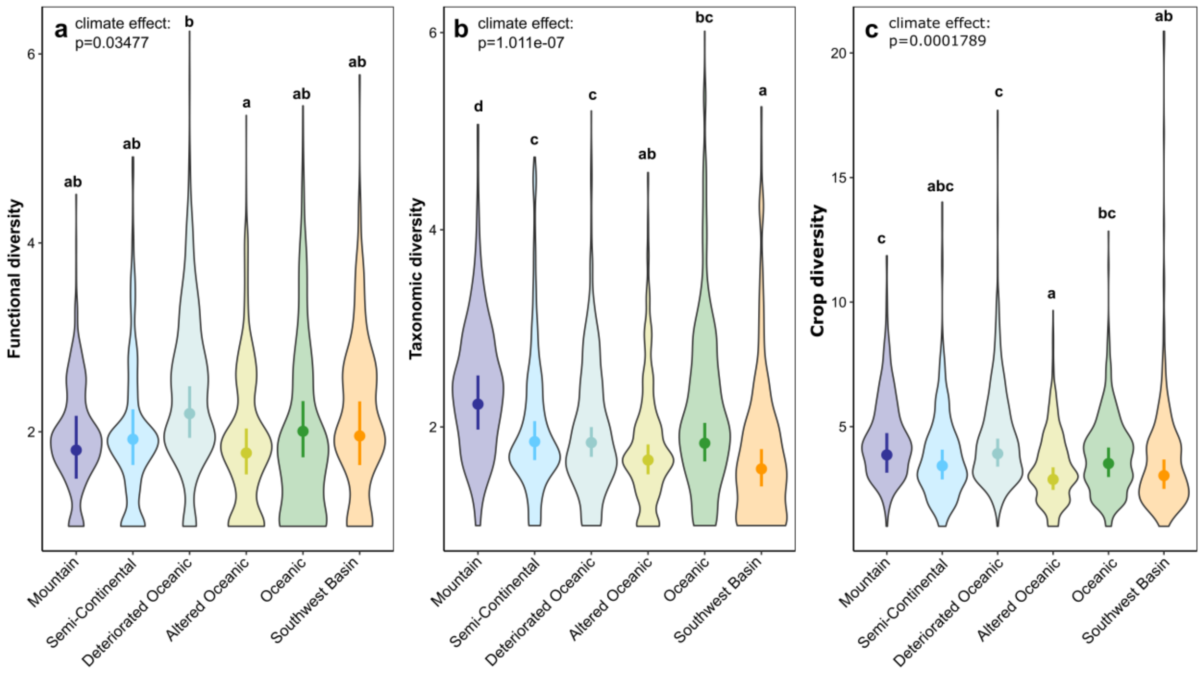


**Supplementary Figure 3: Violin plots highlighting the effect of climatic regions on crop diversity indicators. a: Functional diversity, b: Taxonomic diversity and c: Species diversity (i.e. Functional x Taxonomic diversity)**. Violin plots were created using the observed data. Points represent estimated means and lines associated 95% Wald confidence intervals (computed using the delta method) obtained from a generalized linear mixed effect model (A: model 1, B: model 2 and C: model 3 Supplementary Table 1). Within each graph, violin plots sharing the same letter are not significantly different at $p < 0.05$ based on a set of two-tailed Wald tests, which assess whether or not the pairwise differences in means between climatic regions are different from zero. Multiple comparisons were adjusted using the false discovery rate method. 3761 cropping system described at a given time point (time point referring to either the two-to-three-year average provided by farmers upon entry in the network or the subsequent annual descriptions) were available for all three charts.

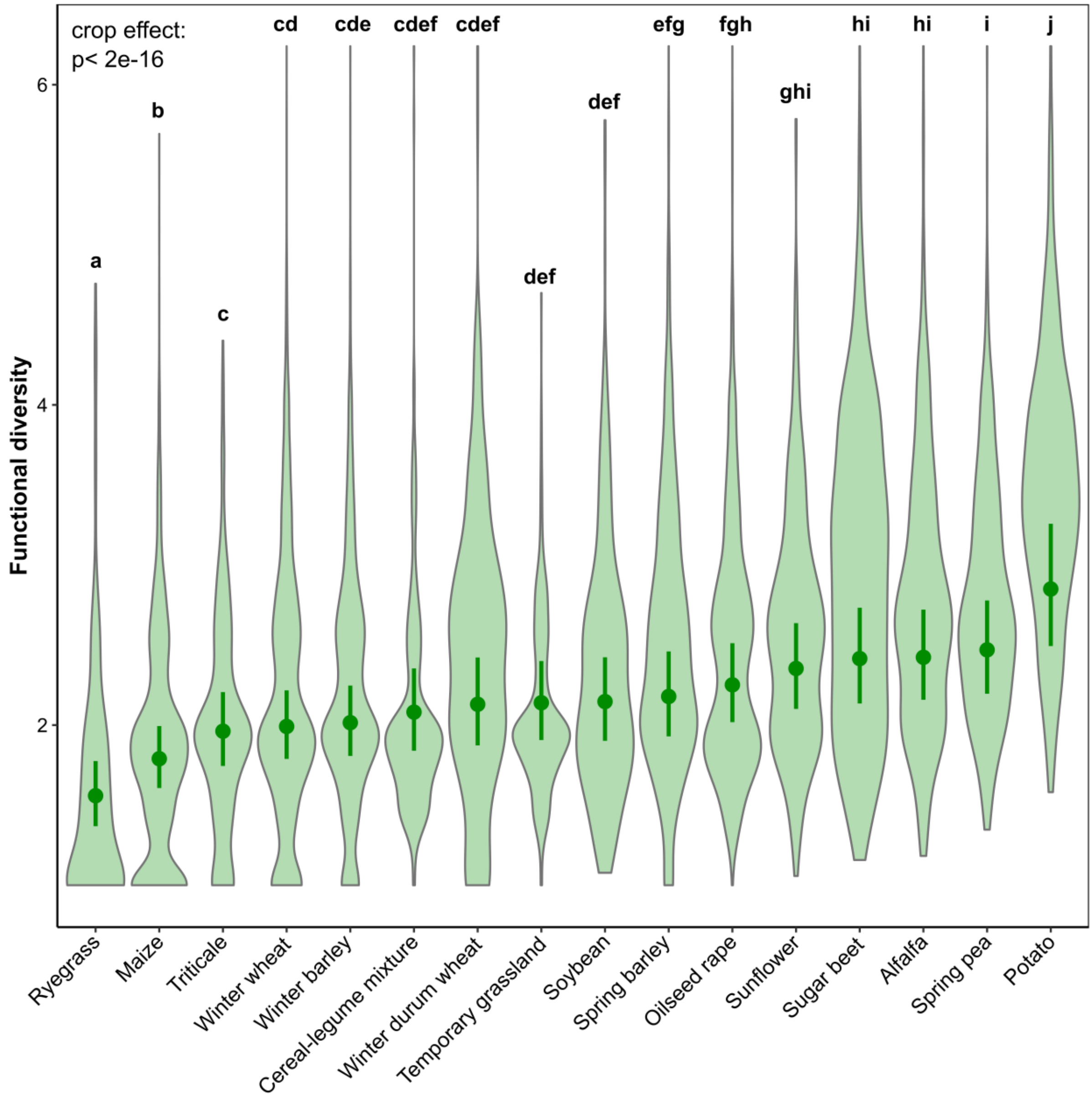


**Supplementary Figure 4: Violin plots highlighting the effect of the 16 main crops on crop functional diversity of the cropping system in which they are included.** Violin plots were created using the observed data. Points represent estimated marginal means and lines associated 95% Wald confidence intervals (computed using the delta method) obtained from a generalized linear mixed effect model (model 4, Supplementary Table 1). The effect of crop was averaged (or "marginalized") across climatic regions. Violin plots sharing the same letters are not significantly different at $p < 0.05$ based on a set of two-tailed Wald tests, which assess whether or not the pairwise differences in crop means are different from zero. Multiple comparisons were adjusted using the false discovery rate method. 3761 cropping system described at a given time point (time point referring to either the two-to-three-year average provided by farmers upon entry in the network or the subsequent annual descriptions) were available for this graph.

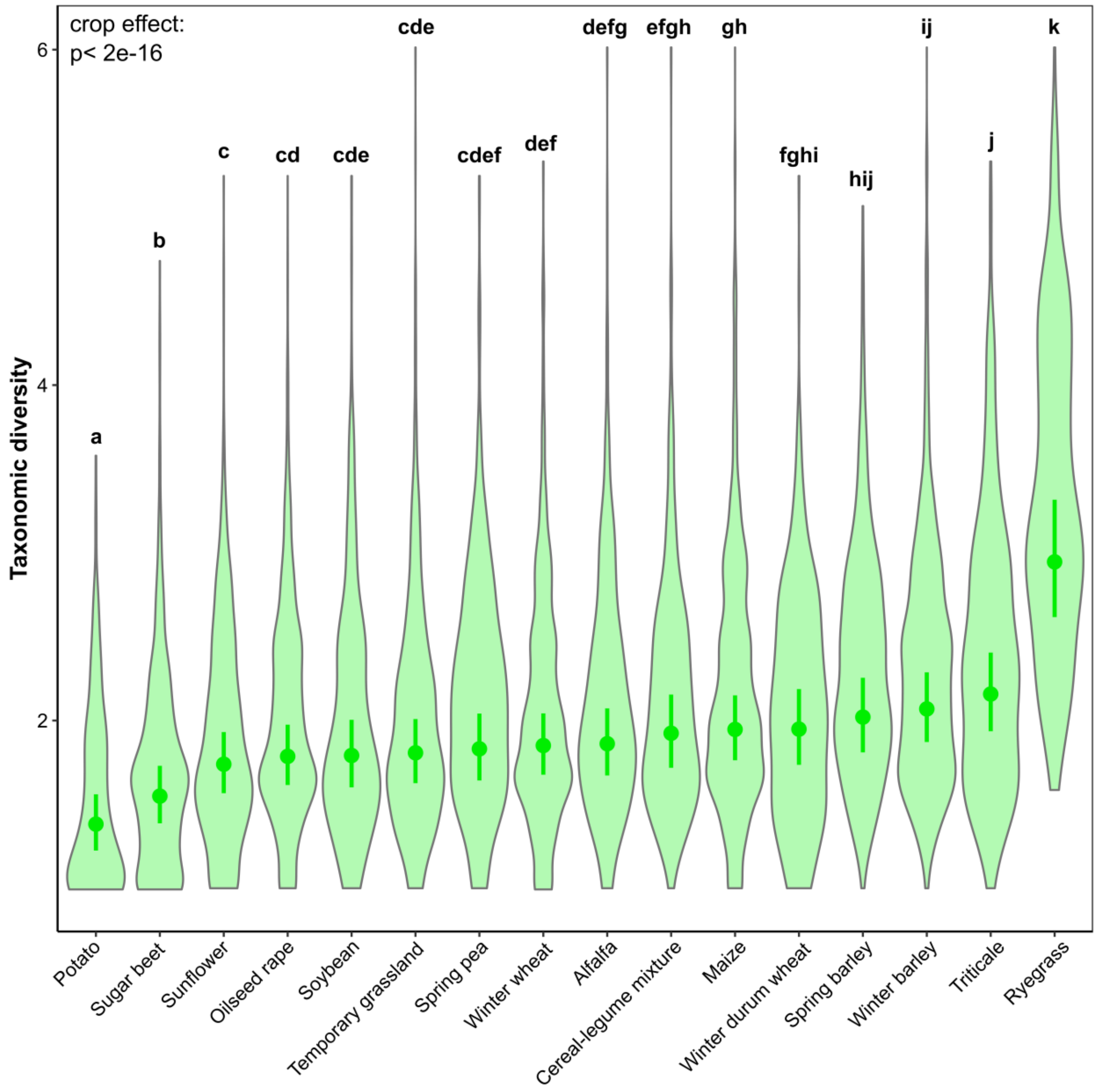


**Supplementary Figure 5: Violin plots highlighting the effect of the 16 main crops on crop x=taxonomic diversity of the cropping systems in which they are included.** Violin plots were created using the observed data. Points represent estimated marginal means and lines associated 95% Wald confidence intervals (computed using the delta method) obtained from a generalized linear mixed effect model (model 5, Supplementary Table 1). The effect of crop was averaged (or "marginalized") across climatic regions. Violin plots sharing the same letters are not significantly different at $p < 0.05$ based on a set of two-tailed Wald tests, which assess whether or not the pairwise differences in crop means are different from zero. Multiple comparisons were adjusted using the false discovery rate method. 3761 cropping system described at a given time point were available for this graph.

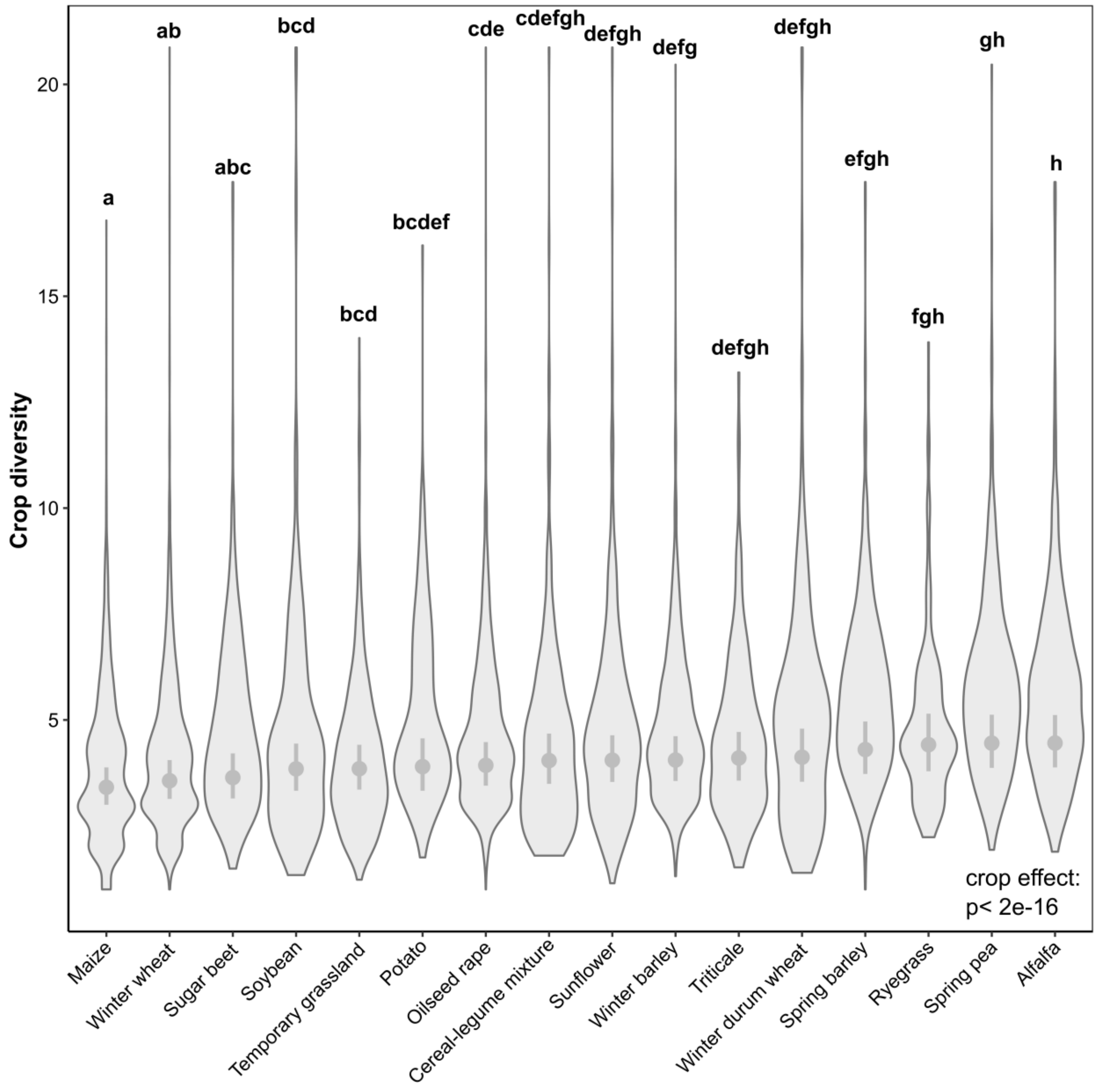


**Supplementary Figure 6: Violin plots highlighting the effect of the 16 main crop on crop species diversity of the cropping systems in which they are included.** Violin plots were created using the observed data. Points represent estimated marginal means and lines associated 95% Wald confidence intervals (computed using the delta method) obtained from a generalized linear mixed effect model (model 6, Supplementary Table 1). The effect of crop was averaged (or "marginalized") across climatic regions. Violin plots sharing the same letters are not significantly different at $p < 0.05$ based on a set of two-tailed Wald tests, which assess whether or not the pairwise differences in crop means are different from zero. Multiple comparisons were adjusted using the false discovery rate method. 3761 cropping system described at a given time point (time point referring to either the two-to-three-year average provided by farmers upon entry in the network or the subsequent annual descriptions) were available for this graph.

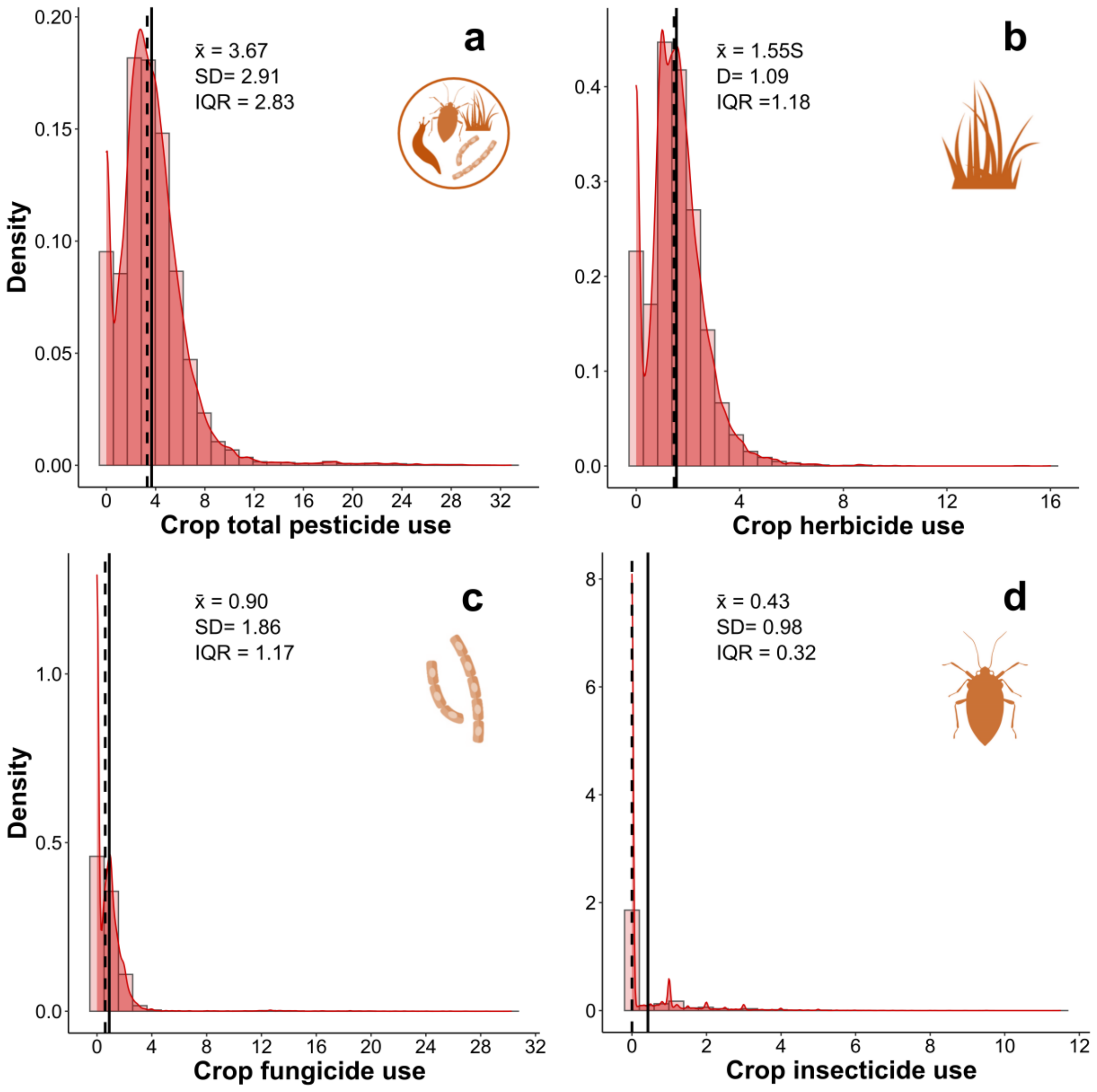


**Supplementary Figure 7: Probability density of crop pesticide use. a: Total pesticide use, b: Herbicide use, c: Fungicide use and d: Insecticide use across the 16 main crops considered in this study.** Pesticide use of crops was assessed with the Treatment Frequency Index (TFI) which quantifies the number of applications at the full recommended dose. One data point (N=14456) represents a TFI value for one crop in a given cropping system at a given time point (time point referring to either the two-to-three-year average provided by farmers upon entry in the network or the subsequent annual descriptions). If multiple fields were represented by the same crop in a given cropping system at a given time point TFI values for this crop were averaged across fields. Kernel bandwidth was chosen using Silverman's rule-of-thumb method. Probability density was estimated with gaussian kernel method and describes the probability densities associated with TFI values. Vertical solid lines represent the mean. Vertical dashed lines represent the median. x̄ = mean; SD= standard deviation; IQR= Interquartile range

Total pesticide use (± standard deviation, SD) averaged 3.7±2.9 across all 14456 observations (i.e. one crop of a given cropping system at a given time point) of the 16 main crops of the dataset, and 1.6±1.1 for herbicides, 0.9±1.9 for fungicides, and 0.4±1 for insecticides. Herbicides, fungicides, and insecticides represented, on average (±SD), 49±25, 19±18, and 8.5±16 % of total pesticide use, respectively. Coefficients of variation (CV) highlighted higher variability in fungicide (210%) and insecticide (230%) use than in herbicide use (70%).

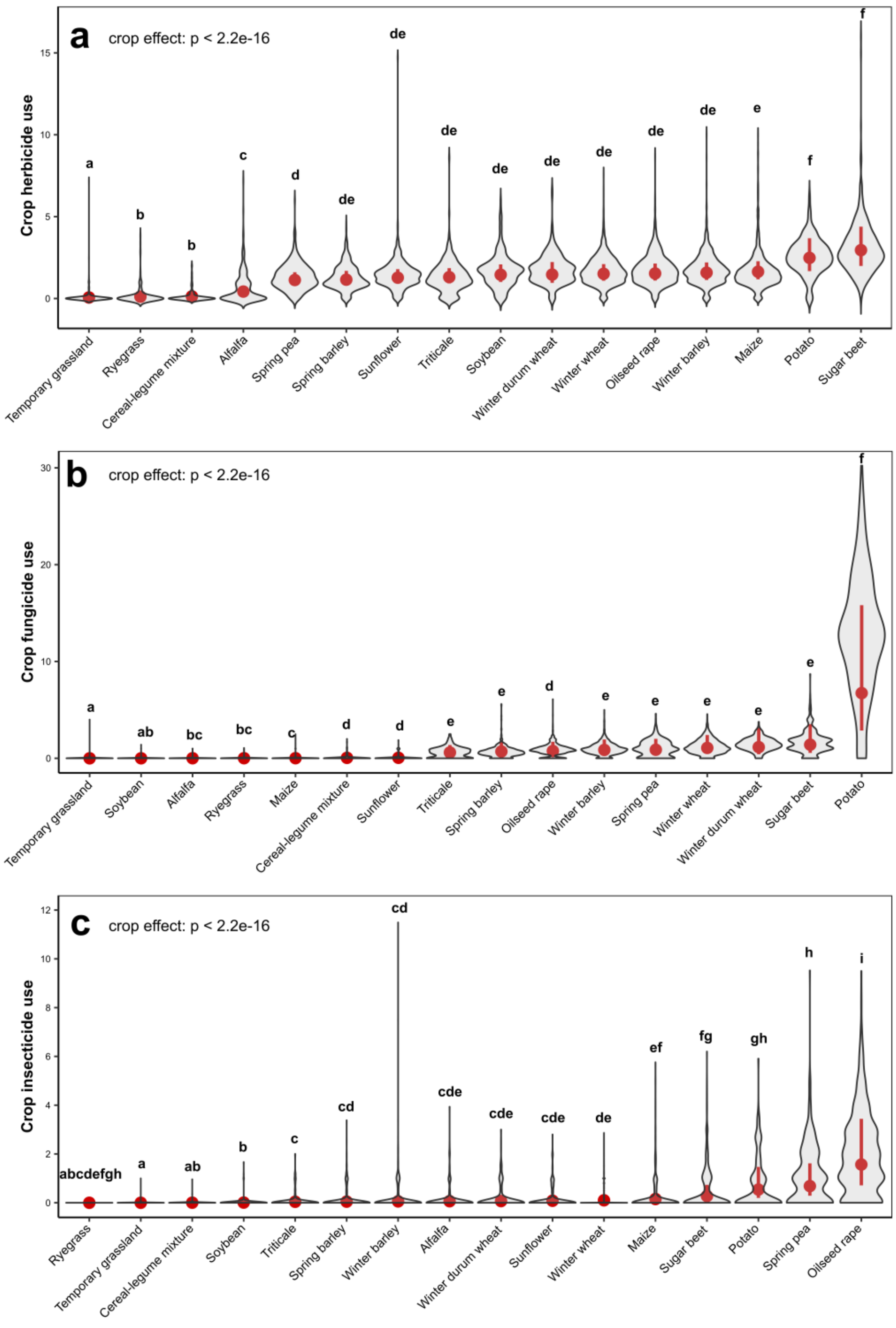


**Supplementary Figure 8: Violin plots highlighting the effect of the 16 main crops on a: herbicide use, b: fungicide use and c: insecticide use.** Pesticide use of crops was assessed with the Treatment Frequency Index (TFI) which quantifies the number of applications at the full recommended dose. Violin plots were created using the observed data. Red points represent estimated marginal means and red lines associated 95% Wald confidence intervals (computed using the delta method) obtained from a generalized linear mixed effect model (A: model 8, B: model 9, C: model 10 Supplementary Table 1). Within each graph, violin plots sharing the same letters are not significantly different at p< 0.05 based on a set of two-tailed Wald tests, which assess whether or not the pairwise differences in crop means are different from zero. Multiple comparisons were adjusted using the false discovery rate method. 14456 observations (i.e. one crop of a cropping system at a given time point - time point referring to either the two-to-three-year average provided by farmers upon entry in the network or the subsequent annual descriptions) were available for this graph. If multiple fields were represented by the same crop in a given cropping system at a given time point, TFI values for this crop were averaged across fields.

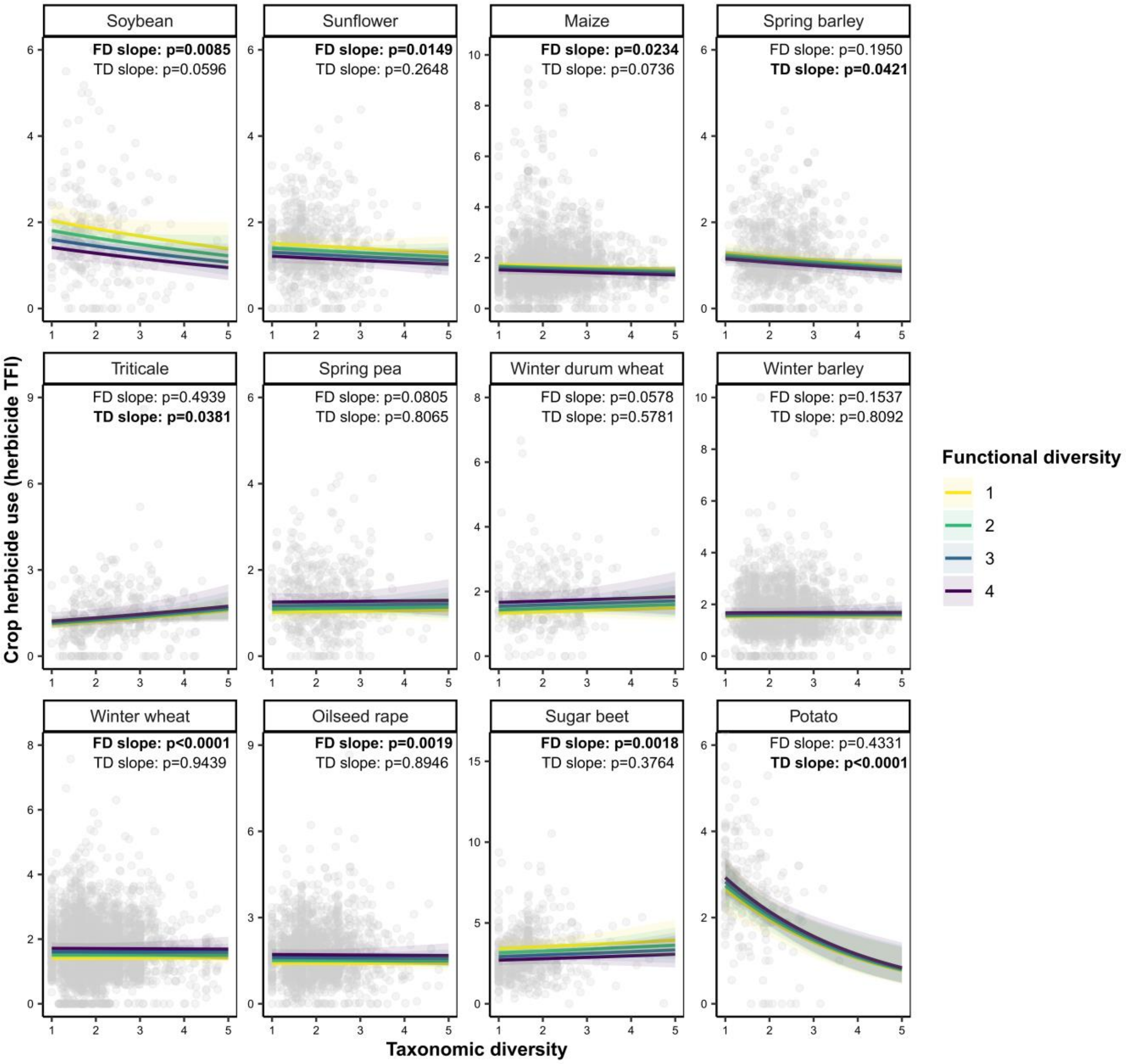


**Supplementary Figure 9: Relationship between crop diversity (taxonomic and functional) and crop herbicide use for 12 main crops. Crop** herbicide use was assessed using the Herbicide Treatment Frequency Index which quantifies the number of herbicide applications at the full recommended dose. Crops with null to low herbicide use (i.e. less than 0.5) were not included in this analysis. One data point (N=12812) represents a crop in a given cropping system at a given time point (either the two-to-three-year average provided by farmers upon entry in the network or the subsequent annual descriptions). When a given crop was grown over multiple fields of a given cropping system at a given time point, values were averaged. Regression lines represent population-level predictions (i.e. do not account for random effects) based on a Tweedie mixed effect model (model 12 supplementary Table 1). Predictions were averaged (or "marginalized") across climatic regions. The upper and lower limits represent Wald 95% confidence intervals (CI) obtained using the delta method. Slopes of Functional (FD slope) and Taxonomic diversity (TD slope) were tested against zero for each crop using a two-tailed Wald test. P-values (p) are provided and written in bold for slopes significantly different from zero at $p<0.05$.

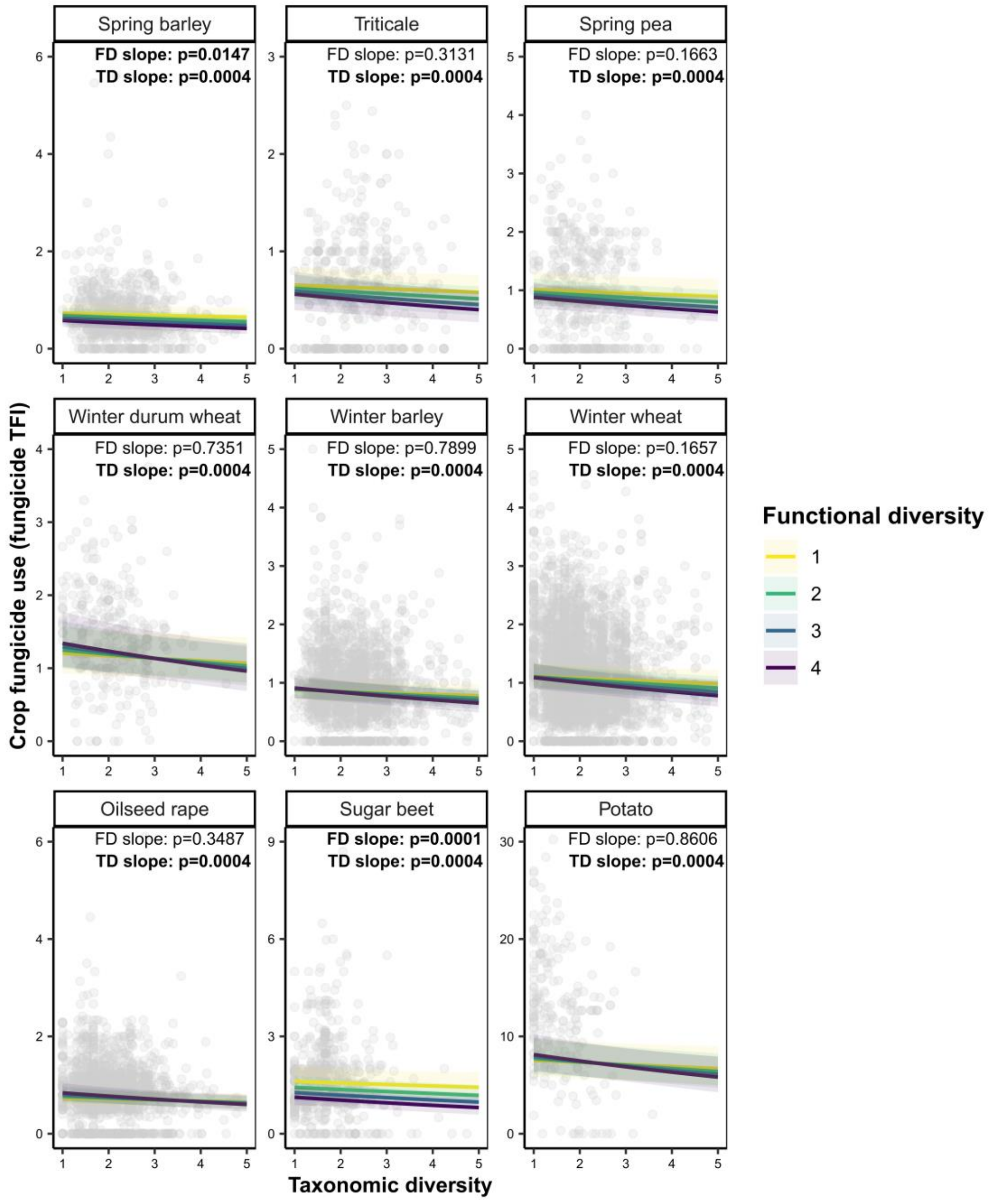


**Supplementary Figure 10: Relationship between crop diversity (taxonomic and functional) and crop fungicide use for 9 main crops. Crop** Fungicide use was assessed with the Fungicide Treatment Frequency Index which quantifies the number of fungicide applications at the full recommended dose. Crops with null to low fungicide use (i.e. less than 0.1) were not included in this analysis. One data point (N=9419) represents a crop in a given cropping system at a given time point (either the two-to-three-year average provided by farmers upon entry in the network or the subsequent annual descriptions). When a given crop was grown over multiple fields of a given cropping system at a given time point, values were averaged. Regression lines represent population-level predictions (i.e. do not account for random effects) based on a Tweedie mixed effect model (model 14 supplementary Table 1). Predictions were averaged (or "marginalized") across climatic regions. The upper and lower limits represent Wald 95% confidence intervals (CI) obtained using the delta method. Slopes of Functional (FD slope) and Taxonomic diversity (TD slope) were tested against zero for each crop using a two-tailed Wald test. P-values (p) are provided and written in bold for slopes significantly different from zero at $p<0.05$.

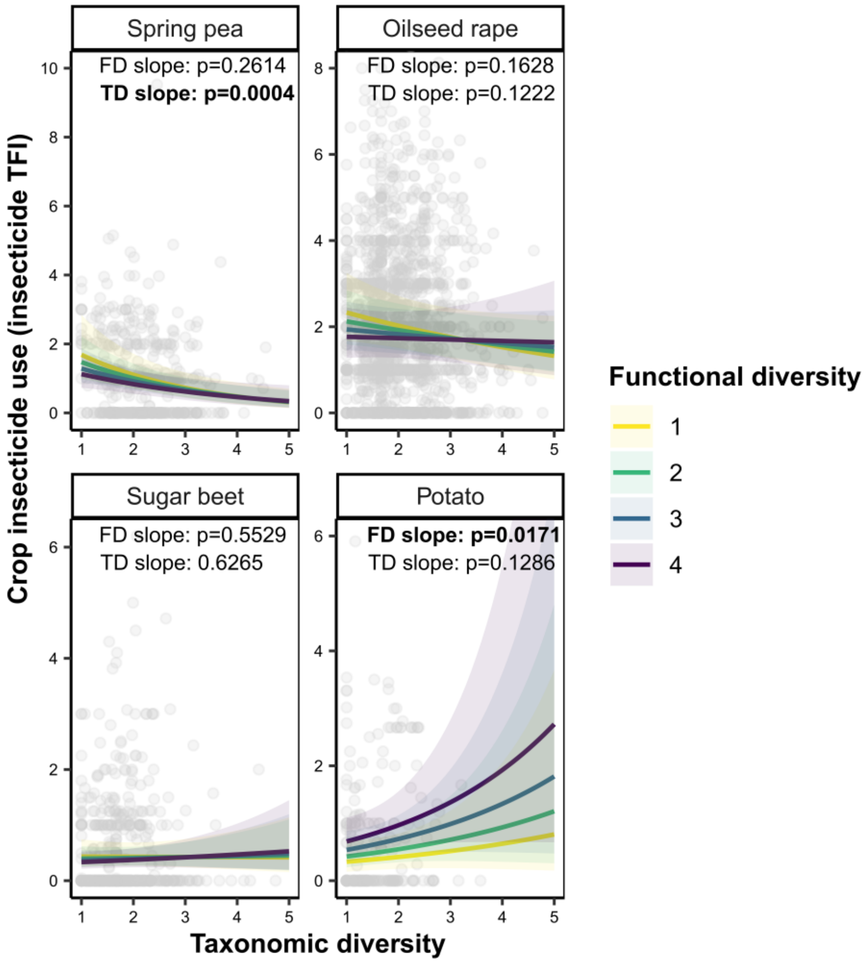


**Supplementary Figure 11: Relationship between crop diversity (taxonomic and functional) and crop insecticide use for 2 main crops.** Insecticide use of crops was assessed with the Insecticide Treatment Frequency Index which quantifies the number of insecticide applications at the full recommended dose. Crops with null to low insecticide use (i.e. less than 0.2) were not included in this analysis. One data point (N=2950) represents a crop in a given cropping system at a given time point (either the two-to-three-year average provided by farmers upon entry in the network or the subsequent annual descriptions). When a given crop was grown over multiple fields of a given cropping system at a given time point, values were averaged. Regression lines represent population-level predictions (i.e. do not account for random effects) based on a Tweedie mixed effect model (model 15 supplementary Table 1). Predictions were averaged (or "marginalized") across climatic regions. The upper and lower limits represent Wald 95% confidence intervals (CI) obtained using the delta method. Slopes of Functional (FD slope) and Taxonomic diversity (TD slope) were tested against zero for each crop using a two-tailed Wald test. P-values (p) are provided and written in bold for slopes significantly different from zero at $p<0.05$.

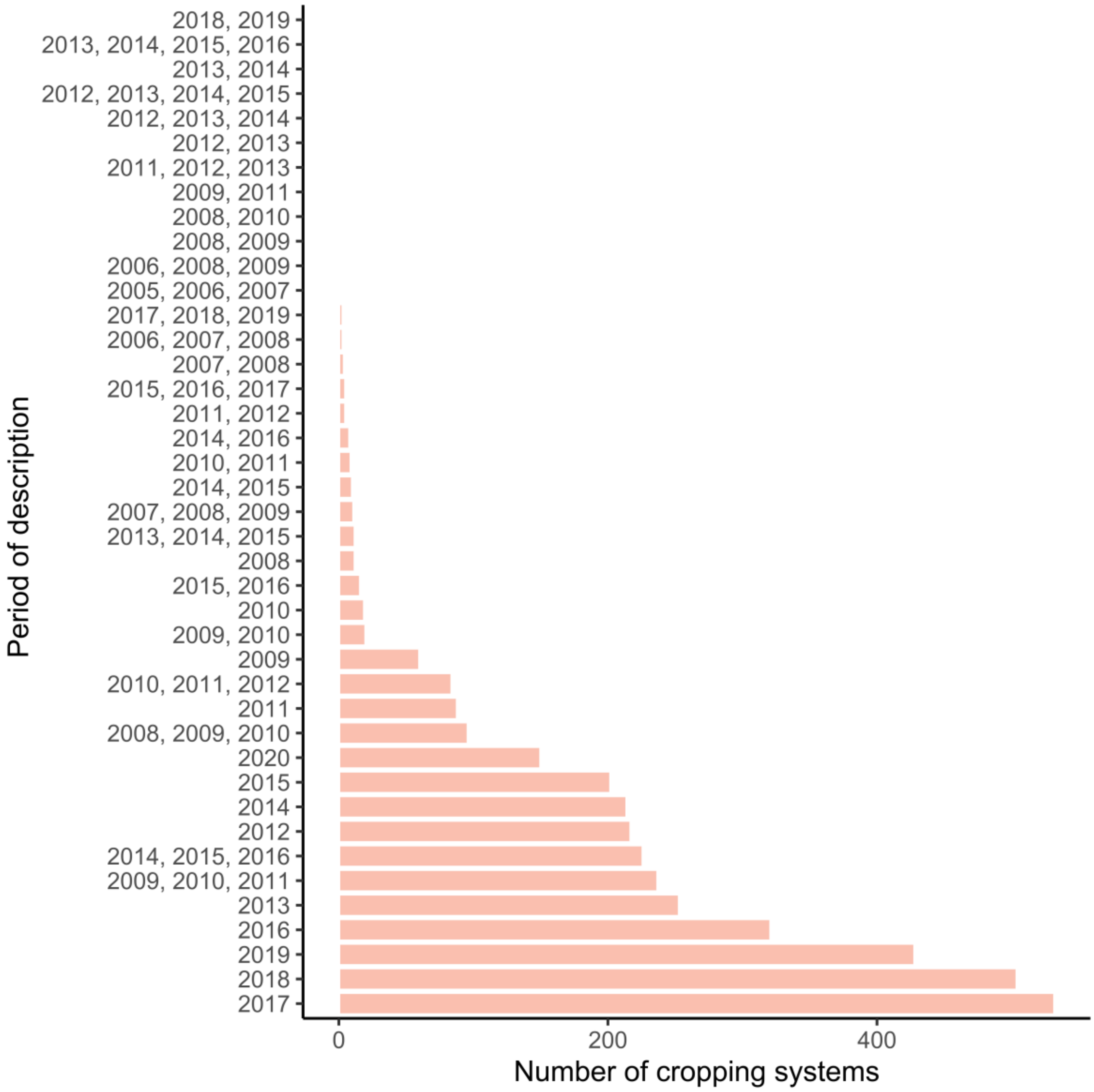


**Supplementary Figure 12: Number of cropping system per period of description.** Description periods encompassing two to three years correspond to farmers' questionnaires upon entry in the network.

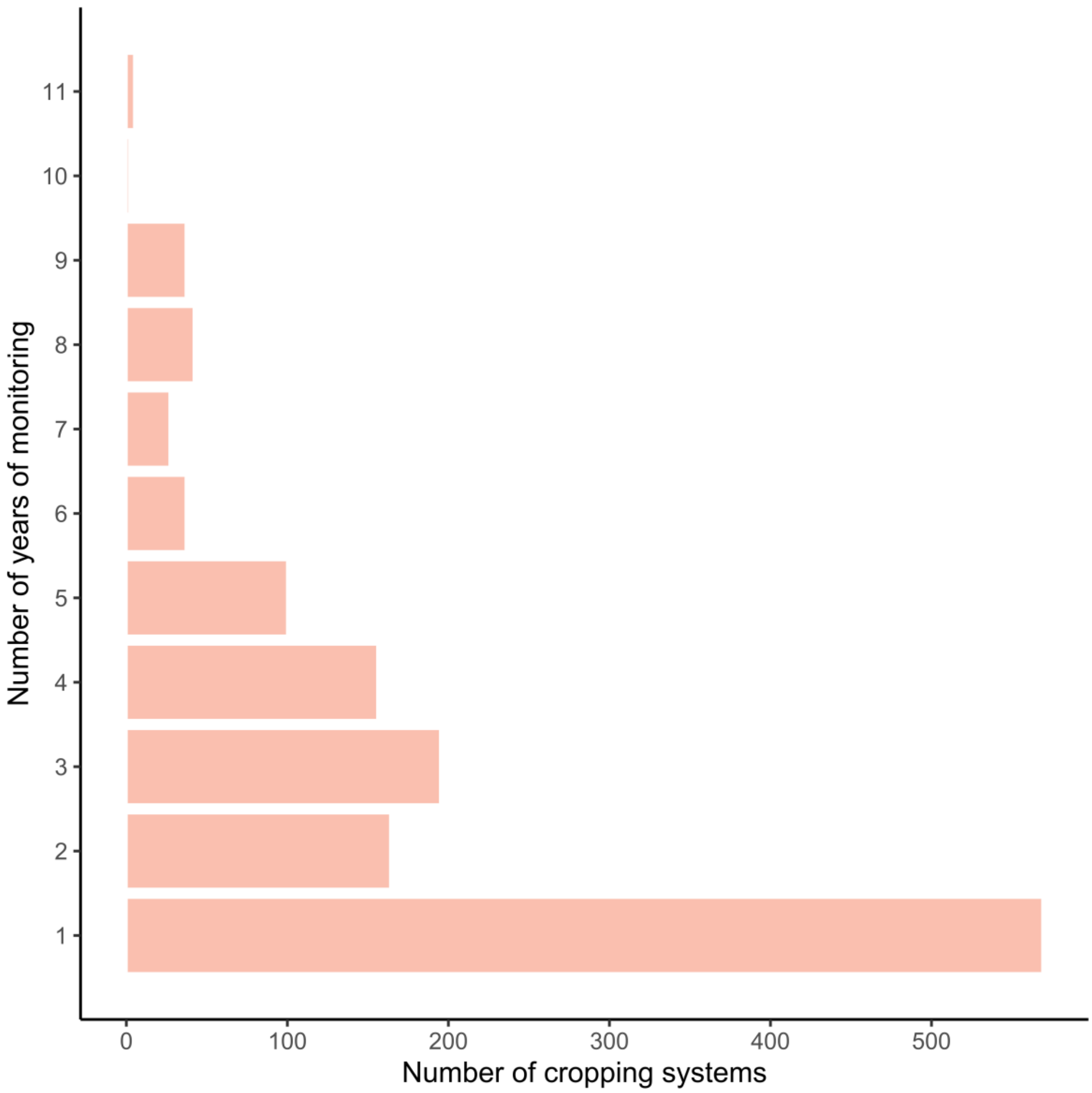


**Supplementary Figure 13: Distribution of monitoring length across all cropping systems.**

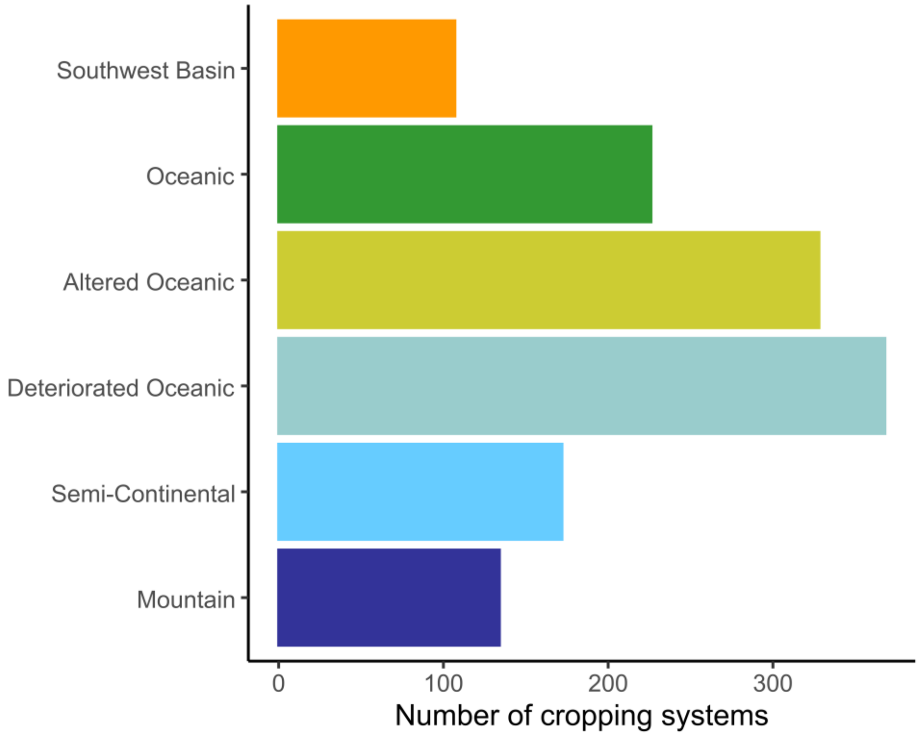


**Supplementary Figure 14: Number of cropping systems in each of the six French climatic regions.**

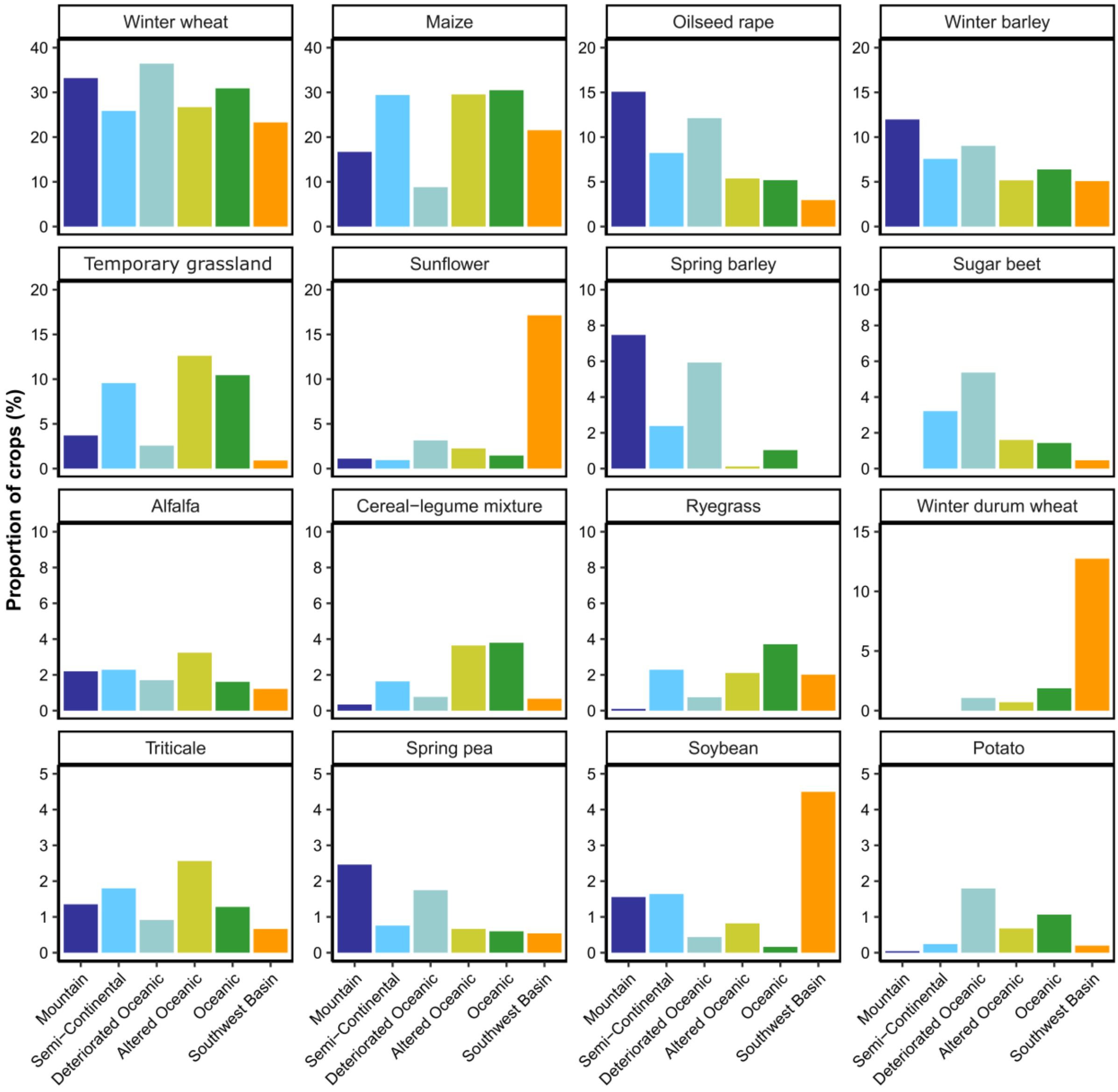


**Supplementary Figure 15: Average proportion of the 16 main crops within each of the six French climatic regions**.

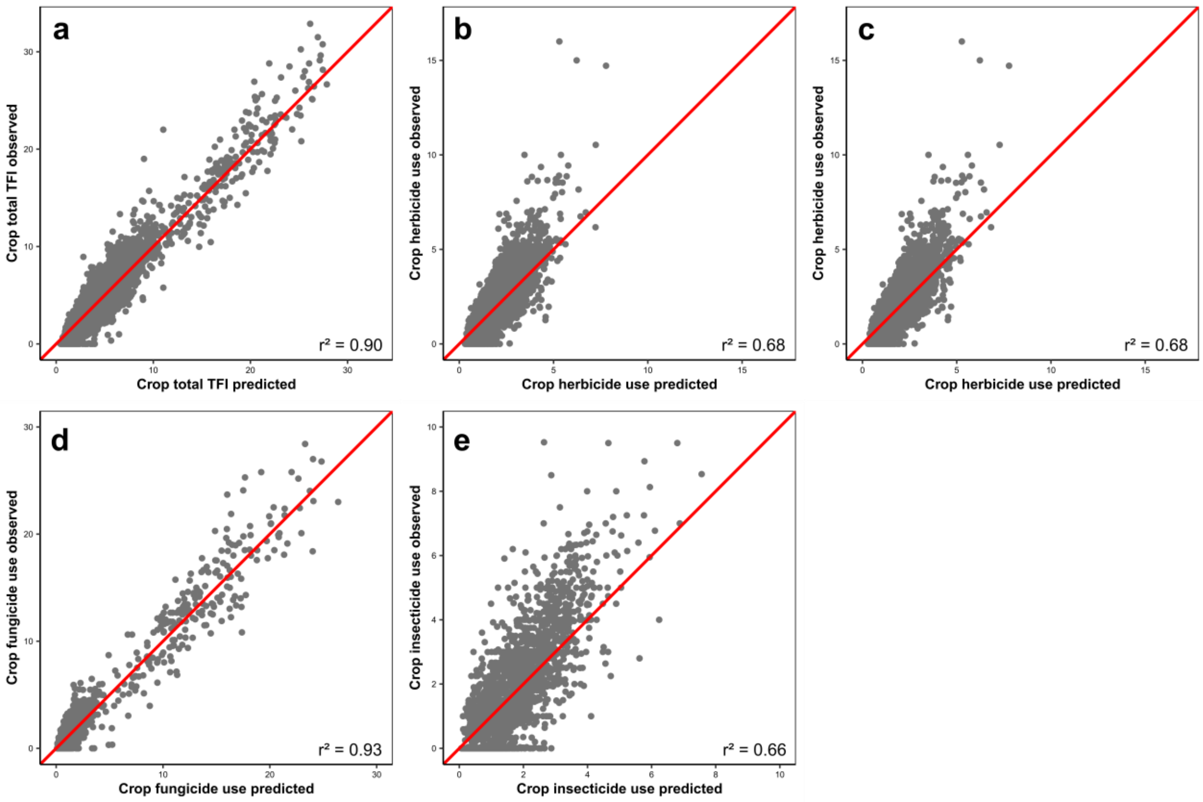


**Supplementary Figure 16: Graphs of fitted vs. observed values for a: crop total pesticide use** (model 11 Supplementary Table 1)**, b: crop herbicide use** (model 12)**, c: crop herbicide use** (model 13)**, d: crop fungicide use** (model 14) **and e: crop insecticide use** (model 15). Values provided at the bottom right of each graph ($r^2$) represent the squared Pearson correlation coefficient between fitted and observed values.
