# Supplementary material for: Fostering temporal crop diversification to reduce pesticide use
Source: Nat Commun. 2023 Nov 16;14:7416. doi: 10.1038/s41467-023-43234-x (PMC10654721; doi:10.1038/s41467-023-43234-x)
Supplement: Supplementary file 3 — Reporting Summary [file 41467_2023_43234_MOESM3_ESM.pdf]

nature portfolio

Corresponding author(s): Maé GUINET

Last updated by author(s): Oct 19, 2023

Reporting Summary

Nature Portfolio wishes to improve the reproducibility of the work that we publish. This form provides structure for consistency and transparency in reporting. For further information on Nature Portfolio policies, see our Editorial Policies and the Editorial Policy Checklist.

Statistics

For all statistical analyses, confirm that the following items are present in the figure legend, table legend, main text, or Methods section.

n/a	Confirmed	
☐	☒	The exact sample size (*n*) for each experimental group/condition, given as a discrete number and unit of measurement
☐	☒	A statement on whether measurements were taken from distinct samples or whether the same sample was measured repeatedly
☐	☒	The statistical test(s) used AND whether they are one- or two-sided<br>*Only common tests should be described solely by name; describe more complex techniques in the Methods section.*
☐	☒	A description of all covariates tested
☐	☒	A description of any assumptions or corrections, such as tests of normality and adjustment for multiple comparisons
☐	☒	A full description of the statistical parameters including central tendency (e.g. means) or other basic estimates (e.g. regression coefficient) AND variation (e.g. standard deviation) or associated estimates of uncertainty (e.g. confidence intervals)
☐	☒	For null hypothesis testing, the test statistic (e.g. *F*, *t*, *r*) with confidence intervals, effect sizes, degrees of freedom and *P* value noted<br>*Give P values as exact values whenever suitable.*
☒	☐	For Bayesian analysis, information on the choice of priors and Markov chain Monte Carlo settings
☐	☒	For hierarchical and complex designs, identification of the appropriate level for tests and full reporting of outcomes
☐	☒	Estimates of effect sizes (e.g. Cohen's *d*, Pearson's *r*), indicating how they were calculated

Our web collection on statistics for biologists contains articles on many of the points above.

Software and code

Policy information about availability of computer code

Data collection	No software was used for data collection.
Data analysis	All data analysis were carried out with the R software version 4.3.1. The R script use to analyze the data and generate the figures was deposited in the Data INRAE repository and is available at the following link: https://doi.org/10.57745/NHWIQN

For manuscripts utilizing custom algorithms or software that are central to the research but not yet described in published literature, software must be made available to editors and reviewers. We strongly encourage code deposition in a community repository (e.g. GitHub). See the Nature Portfolio guidelines for submitting code & software for further information.

Data

Policy information about availability of data

All manuscripts must include a data availability statement. This statement should provide the following information, where applicable:

- Accession codes, unique identifiers, or web links for publicly available datasets
- A description of any restrictions on data availability
- For clinical datasets or third party data, please ensure that the statement adheres to our policy

The data used in the present article were extracted from the Agrosyst database. The Agrosyst database compiles description and performance of all the cropping systems (i.e. set of plots within a farm which followed the same crop rotation, countrains and decision rules) monitored within the French National DEPHY Network. The extraction comprises all cropping systems monitored before 2021 (excluded). The datasets that support the findings of this study were deposited in the Data

INRAE repository and are fully accessible at https://doi.org/10.57745/NHWIQN. The E-phy online database provided by the French Ministry of Agriculture was used in this study to extract reference doses of commercial pesticide products.

Research involving human participants, their data, or biological material

Policy information about studies with human participants or human data. See also policy information about sex, gender (identity/presentation), and sexual orientation and race, ethnicity and racism.

Reporting on sex and gender	not concerned
Reporting on race, ethnicity, or other socially relevant groupings	not concerned
Population characteristics	not concerned
Recruitment	not concerned
Ethics oversight	not concerned

Note that full information on the approval of the study protocol must also be provided in the manuscript.

Field-specific reporting

Please select the one below that is the best fit for your research. If you are not sure, read the appropriate sections before making your selection.

☐ Life sciences ☐ Behavioural & social sciences ☒ Ecological, evolutionary & environmental sciences

For a reference copy of the document with all sections, see nature.com/documents/nr-reporting-summary-flat.pdf

Ecological, evolutionary & environmental sciences study design

All studies must disclose on these points even when the disclosure is negative.

Study description	The objectif of this study was to assessed the effect of temporal crop diversification (assessed through functional diversity, taxonomic diversity, crop diversity, effective number of sowing periods and cover crop frequency) on pesticide use in 16 main crops.
Research sample	1334 cropping systems, were implemented in arable crops and/or mixed farming spanned accross six climatic regions of the French National Metropolitan Territory (no data was present on cropping systems located in the central mediterranean as field crops are poorly represented in this region)
Sampling strategy	All cropping systems implemented in arable crops and/or mixed farming monitored within the French National DEPHY network. Only volunteer farmers are present in the network. All fully informed (i.e. complete) crop rotation were used.
Data collection	Local network engineers (roughly 150) monitored around 10 farms each and gathered data from volunteer farmers (through surveys and/or by extracting data present in farmer's softwares). The identity of the crop and the management practices (date, dose, commercial product, agricultural equipment,...) implemented on each plot of a cropping system described at a given time point (time point referring to either the two-to-three-year average provided by farmers upon entry in the network or the subsequent annual descriptions)
Timing and spatial scale	Cropping systems (i.e. a set of plots within a farm which followed the same crop rotation, countrains and decision rules) were described between 2008 and 2020. Only a small proportion of cropping systems were monitored for more than five years and temporal diversification was here assessed through time for space substitution. We assumed that crop diversity described for a given cropping system at a given time point was representative of the crop rotational diversity. Spatial scale : French metropolitan area excluding central mediterranean (as field crops are poorly represented in this region)
Data exclusions	Organic cropping systems were excluded because synthetic pesticides are prohibited in such systems and hence, no variability could be analyzed. Variability of pesticide use can be analyzed/interpreted as a performance indicator in conventional systems but not in organic systems. Pure grassland cropping systems were discarded
Reproducibility	This study is based on nation wide monitoring data and hence cannot be replicated as experimental designs.
Randomization	This study is based on nation wide monitoring data and hence the structure of the dataset was not a priori defined.
Blinding	Not relevant. Data was nevertheless anonymized and GPS data limited to the city level.

Did the study involve field work? ☐ Yes ☒ No

Reporting for specific materials, systems and methods

We require information from authors about some types of materials, experimental systems and methods used in many studies. Here, indicate whether each material, system or method listed is relevant to your study. If you are not sure if a list item applies to your research, read the appropriate section before selecting a response.

Materials & experimental systems

n/a	Involved in the study
☒	☐ Antibodies
☒	☐ Eukaryotic cell lines
☒	☐ Palaeontology and archaeology
☒	☐ Animals and other organisms
☒	☐ Clinical data
☒	☐ Dual use research of concern
☒	☐ Plants

Methods

n/a	Involved in the study
☒	☐ ChIP-seq
☒	☐ Flow cytometry
☒	☐ MRI-based neuroimaging
